# TP53INP2 Contributes to TGF-β2-Induced Autophagy during the Epithelial–Mesenchymal Transition in Posterior Capsular Opacification Development

**DOI:** 10.3390/cells11152385

**Published:** 2022-08-02

**Authors:** Yilei Cui, Hao Yang, Silu Shi, Xiyuan Ping, Sifan Zheng, Xiajing Tang, Xiaoning Yu, Xingchao Shentu

**Affiliations:** 1 Eye Center, The Second Affiliated Hospital, Zhejiang University School of Medicine, Hangzhou 310009, China; 12018532@zju.edu.cn (Y.C.); 12018535@zju.edu.cn (H.Y.); silu46@zju.edu.cn (S.S.); pingxiyuan@126.com (X.P.); xiajingtang@zju.edu.cn (X.T.); yxnzju@163.com (X.Y.); 2 Zhejiang Provincial Key Lab of Ophthalmology, Hangzhou 310009, China; 3 GKT School of Medical Education, King’s College London, London WC2R 2LS, UK; sifan.zheng@kcl.ac.uk

**Keywords:** TP53INP2, TGF-β2, autophagy, posterior capsule opacification, epithelial–mesenchymal transition

## Abstract

Background: Posterior capsule opacification (PCO) is the most common complication after cataract surgery, in which increased levels of transforming growth factor-beta 2 (TGF-β2) accelerate PCO formation; however, the pathological mechanisms are not fully understood. This study aims to explore the regulation mechanism of TGF-β2 in PCO formation via its autophagic functions. Methods: The autophagic effect of TGF-β2 was detected by transmission electron microscopy (TEM), Western blotting, and immunofluorescence analysis. The association between autophagy and the epithelial–mesenchymal transition (EMT) was evaluated by qPCR and Western blotting. The transcriptome analysis was used to uncover the molecular mechanism of TGF-β2-induced PCO formation. Results: TGF-β2 specifically promotes autophagy flux in human lens epithelial cells. The activation of autophagy by rapamycin can promote EMT marker synthesis and improve cell migration. However, the inhibition of autophagy by 3-MA attenuates EMT. To uncover the molecular mechanisms, we performed RNA sequencing and found that TGF-β2 elevated tumor protein p53-inducible nuclear protein2 (TP53INP2) expression, which was accompanied by a nuclear-to-cytoplasm translocation. Moreover, the knockdown of TP53INP2 blocked the TGF-β2-induced autophagy and EMT processes, revealing that TP53INP2 plays an important role in TGF-β2-induced autophagy during EMT. Conclusions: Taken together, the results of this study suggested that TP53INP2 was a novel regulator of PCO development by TGF-β2, and notably, TP53INP2, may be a potential target for the pharmacological treatment of PCO.

## 1. Introduction

Posterior capsule opacification (PCO) is the most common complication after cataract surgery, causing decreased vision in 20–40% of elderly patients [1] and 100% of pediatric patients [1,2]. While the specific pathological mechanisms are not fully understood, it is widely accepted that the epithelial–mesenchymal transition (EMT) process is a pivotal cause. During the EMT, residual lens epithelial cells lose adhesion and connexin. The epithelial cells are subsequently trans-differentiated into mesenchymal cells and rapidly migrated to the posterior capsule [3,4]. Multiple cytokines are reported to trigger EMT, including FGF-2, EGF, and TGF-β2 (transforming growth factor-β2) [5,6]. TGF-β2 levels, as a major inducer of EMT, are significantly increased after cataract surgery and accelerate PCO formation. Currently, there is no ideal pharmacological treatment for PCO.

Autophagy is an evolutionally conserved degradative process that engulfs damaged, degenerative or aging proteins and organelles into phagophores, which mature into double-membrane autophagosomes and then fuse with lysosomes for degradation [7]. Basal autophagy serves as a quality control system and is required for maintaining cellular homeostasis [8]. Consequently, imbalanced autophagy may induce cancer, neurodegenerative diseases, and ocular disorders, including cataracts [9]. However, the precise effect of autophagy on the lens is an open area of inquiry. Our previous research had found that rapamycin (rapa), an activator of autophagy, effectively rescued lens opacities in GJA8^−/−^ zebrafish [10]. Conversely, hyperactive autophagy may result in cell viability deprivation and death [11]. These studies confirmed the important role of autophagy in regulating lens homeostasis. Currently, there is accumulating evidence that autophagy is regarded as a crucial regulator of EMT in some tumor cells [12]. However, the roles of TGF-β2, EMT, and autophagy involved in the development of PCO are not fully understood.

In this study, we have reported the autophagic response of human lens epithelial (HLE) cells during the EMT process induced by TGF-β2, as well as the regulation of TP53INP2, to investigate the potential mechanism in the formation of PCO.

## 2. Materials and Methods

### 2.1. RNA Sequencing

The HLE cells were seeded onto 10 cm dishes overnight and then treated with or without TGF-β2 (10 ng/mL) for 24 h. Total RNA was extracted by TRIzol Reagent (Invitrogen, Carlsbad, CA, USA), and the RNA purity was detected by spectrophotometer (NanoDrop). The RNA integrity number (RIN) was measured using the Agilent 2200 TapeStation (Agilent Technologies, Palo Alto, CA, USA) and the sample with RIN ≥ 7 was chosen for sequencing. Subsequently, library preparation and RNA sequencing were performed by RiboBio Co., Ltd. (Guangzhou, China) using sequencing (2 × 150 bp) HiSeq3000.

The detailed information on sequencing was described previously [13], and the differential expression was estimated by DEseq2 v1.16.1 using read counts as input. The correction method of the Benjamini–Hochberg multiple test was utilized. Differentially expressed genes (DEGs) were selected according to the criteria of log2|Fold-change| > 1.2, adjusted *p*-value < 0.05, for further transcriptomic analysis.

### 2.2. Cell Culture, Treatment, and Transfections

Human lens epithelial (HLE) cell lines (SRA01-04, RIKEN Cell Bank, Japan) were maintained in DMEM (Corning, NY, USA) with 20% FBS, (AusgeneX, Brisbane, Queensland, Australia) and a 1% penicillin–streptomycin solution (Gibco, Carlsbad, CA, USA) in a 5% CO_2_ incubator at 37℃. For TGF-β2 stimulation, the cells were seeded onto the 6-well plates overnight and then treated with different doses of TGF-β2 (302-B2, R&D, Emeryville, CA, USA) for 24 h. For rapamycin, 3-methyladenine (3-MA), and Bafilomycin A1(BafA1) treatment, the HLE cells were pre-treated with rapa (200 nM, HY-10219, MCE, NJ, USA), 3-MA (10 Mm, HY-19312, MCE), and bafA1 (50 nM, 54645S, CST, Boston, MA, USA) for 4 h before the addition of TGF-β2 and then treated for 24 h. For transfection, a total of 5 × 10^5^ HLE cells were mixed in 200 uL opti-MEM with siTP53INP2 (100 nM) and 7.5 uL Lipofectamine 3000 reagents (Invitrogen, Carlsbad, CA, USA) at 37 °C, following the manufacturer’s instructions. After 24 h of transfection, the silencing efficiency of siRNA was detected by Q-PCR, and the cells were cultured with or without TGF-β2 for another 24 h. The following sequences were used: siTP53INP2 (5-CCGAAACCUCCCUUCUUAATT-3) and NC siRNA (5-UUCUCCGAACGUGUCACGU-3). The RNA oligonucleotides were obtained by RiboBio.

### 2.3. Western Blotting

Western blotting has been described previously [14]. Briefly, the total protein was collected in RIPA buffer and mixed with loading buffer for denaturing, then separated by SDS-PAGE and transferred to PVDF membranes for subsequent incubation with primary antibodies. The antibodies used were as follows: anti-LC3 (3868s, CST), anti-P62 (ab109012, Abcam, Cambridge, MA, USA), anti-ATG5 (9980s, CST), anti-TP53INP2 (ER65701, HUABIO, Hangzhou, China), anti-vimentin (5741, CST), anti-fibronectin (ab2413, Abcam), anti-beclin1 (3738, CST), anti-E-cadherin (ab40772, Abcam), anti-N-cadherin (ab76011, Abcam), and anti-GAPDH (60004-1-Ig, Proteintech, Chicago, IL, USA).

### 2.4. RNA Isolation, qRT–PCR Analysis

Total mRNA was extracted from cells using Trizol reagent. A total of 500 ng of RNA was transcribed into cDNA according to the manufacturer’s protocol (Vazyme Biotech Co., Ltd., Nanjing, China). qRT–PCR was performed in a ChamQ SYBR qPCR Master Mix kit (Q311-02, Vazyme, Nanjing, China) using a 7500 Fast RT-PCR System. The mRNA levels were assessed using the 2−ΔΔCt quantification and the sequence of primers was as follows: LC3: Forward-TCATCAAGATAATTAGAAGGCGCT, AACAATTCTAGAAGAGCTGCATTT; ATG5: Forward-AAAGATGTGCTTCGAGATGTGT, Reverse-CACTTTGTCAGTTACCAACGTCA; Beclin1: Forward-GGGCTCCCGAGGGATGG, Reverse-TTCCTCCTGGGTCTCTCCTG; E-cadherin: Forward—CGAGAGCTACACGTTCACGG, Reverse—GGGTGTCGAGGGAAAAATAGG; N-cadherin: Forward—AGCCAACCTTAACTGAGGAGT, Reverse—GGCAAGTTGATTGGAGGGATG; Fibronectin: Forward—CCGCCGAATGTAGGACAAGA, Reverse—CTGTCAGAGTGGCACTGGTAG.

### 2.5. Immunofluorescence

After washing with PBS, the cells were fixed in 4% paraformaldehyde solution for 15 min and blocked with 10% goat serum albumin for 1 h, followed by incubating with primary antibodies overnight at 4 °C, and then stained with secondary antibodies and DAPI for 1 h at room temperature. The primary and secondary antibodies used were as follows: anti-LC3 (1:300; 3868s, CST), anti-P62 (1:500, ab109012, Abcam), anti-TP53INP2 (1:200, ER65701, HUABIO), Alexa Fluor 488 (1:1000; A28175, Thermo Fisher Scientific, Waltham, MA, USA), and Alexa Fluor 555 (1:1000; A21428, Thermo Fisher Scientific). After washing three times, the cells were observed using a Nikon A1 confocal microscope.

### 2.6. Transmission Electron Microscopy

The cells were collected and fixed in 2.5% glutaraldehyde (SPI Chem, 02608-BA) in PBS at 4 °C overnight. The cells were rinsed by PBS and fixed with 1% OsO4 and 0.1% K3Fe(CN)6. The cells were then dehydrated by a graded concentration of ethanol and embedded in Spurr resin. Ultrathin sections were stained with 2% uranyl acetate and alkaline lead citrate and observed using a Hitachi Model H-7650 TEM.

### 2.7. Wound Healing Assays

The cells were cultured in two 6 cm dishes and transfected with TP53INP2 siRNA and NC siRNA for 24 h. Subsequently, the above cells were seeded in a 6-well plate to grow to 90% confluence. The cells were scratched with a 100 μL pipette tip and washed twice using PBS, and then incubated in DMEM containing 2% FBS with or without TGF-β2, rapa, or 3-MA for 48 h. The images were taken by microscope (Olympus) and analyzed by Image J software. The quantitative wound healing area was calculated by the equation: [1 − empty area X h/empty area 0 h] × 100.

### 2.8. Subcellular Fractionation

The cells were harvested with cold PBS and lysed in buffer A (Beyotime Biotechnology, Shanghai, China) containing 1% PMSF on ice for 20 min. The cells were added to buffer B and then centrifuged at 13,000 rpm for 5 min. The resultant supernatant was transferred to a new Eppendorf tube and used as cytosolic fractions. The sediment was then homogenized with the buffer of nuclear protein extraction reagent with 1% PMSF. After 30 min of vortexing, the cells were centrifuged for 10 min at 16,000 rpm and the resultant supernatants were used as nuclear fractions.

### 2.9. Statistical Analysis

The data were analyzed using GraphPad Prism 8.0 software and are presented as means ± SD. Student’s *t*-tests were performed to compare the statistical difference between two groups and one-way ANOVA was conducted to compare the data of multiple groups. A value of *p* < 0.05 was considered significant.

## 3. Results

### 3.1. Transcriptome Analysis Identified the TGF-β2 Involved in Autophagy and EMT Processes in HLE Cells

**Figure 1:**
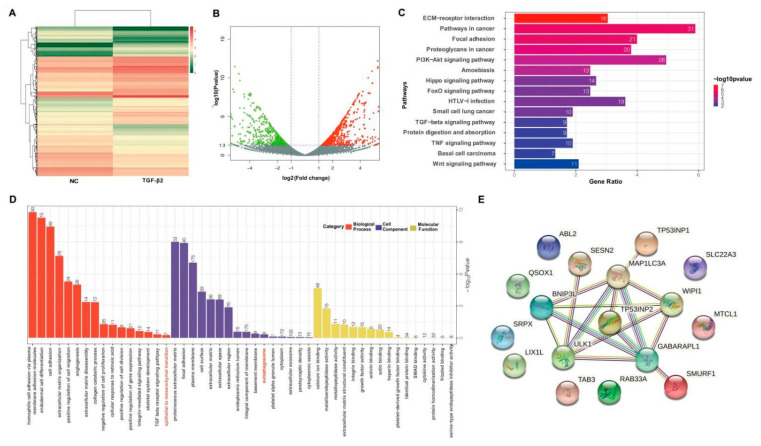
**TGF-β2 is involved in autophagy and the EMT process in HLE cells.** (**A**,**B**) Heatmap (**A**) and volcano plots (**B**) depict the differentially expressed genes in TGF-β2-treated or -untreated HLE cells. The red points, green points, and grey points represent upregulated genes, downregulated genes, and not differentially expressed genes, respectively. (**C**) The enrichment of KEGG pathways in TGF-β2-treated HLE cells vs. untreated cells. (**D**) Gene ontology (GO) analysis of DEGs; the top sixteen GO terms within three main subcategories (BP, CC, and MF) are shown. The significant DEGs of TGF-treated HLE cells are enriched in both autophagy and the EMT. The number on top of the bars represents the number of DEGs annotated to the GO terms. (**E**) Protein–protein interaction (PPI) analysis of upregulated DEGs annotated to GO terms correlated with autophagy.

To reveal the molecular mechanisms of autophagy in TGF-β2-exposed HLE cells, we performed transcriptome profiling to compare the genes involved in PCO development in TGF-β2-treated or -untreated HLE cells. A total of 998 differentially expressed genes (DEGs) were found in the two groups (Figure 1A,B), of which 527 genes were up-regulated and 471 genes were down-regulated (log2|fold-change| > 1.2; adjusted *p*-value < 0.05). The gene ontology (GO) term and Kyoto Encyclopedia of Genes and Genomes (KEGG) pathway were conducted based on up-related DEGs. The top sixteen KEGG pathways are presented in Figure 1C, and most of them were pathways related to EMT and autophagy. For example, (ECM)–receptor interaction, focal adhesion, and the PI3K/Akt pathway were involved in the EMT process and the PI3K/Akt pathway, Hippo pathway, and Wnt pathway were related to autophagy. Similarly, the results of GO analysis showed that the DEGs not only were significantly enriched in the EMT but also in autophagy (Figure 1D). Subsequently, we conducted a protein–protein interaction (PPI) analysis of upregulated DEGs annotated to GO terms correlated with autophagy (Figure 1D). Because it had the highest increase of 4.99-fold, the tumor protein p53 inducible nuclear protein 2 (TP53INP2) was chosen for further exploration as it was related to both autophagy and EMT (Table 1 depicts all significantly changed autophagy genes).

### 3.2. TGF-β2 Promotes Autophagy Flux in HLE Cells

**Figure 2:**
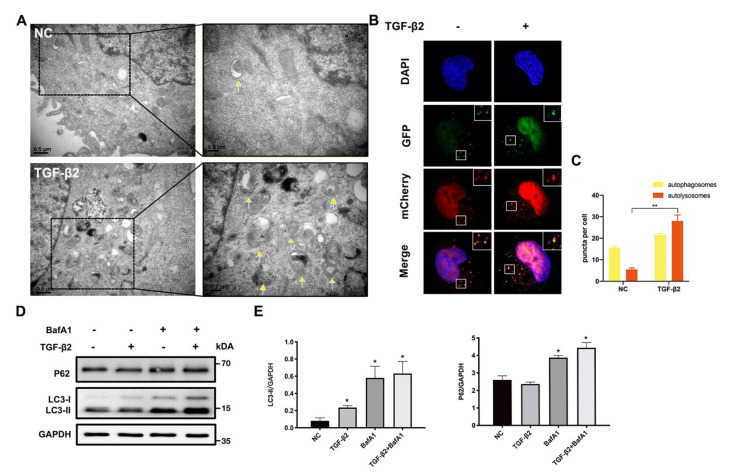
**TGF-β2 promotes autophagy flux in HLE cells.** (**A**) Electron micrographs of TGF-β2 (10 ng/mL)-untreated (NC) or -treated HLE cells. The yellow arrows indicate autophagic vacuoles (AVs). Scale bar: 0.5 μm and 0.2 μm. (**B**) Representative images of GFP-mCherry-LC3 infected HLE cells treated or untreated with TGF-β2 (10 ng/mL) stimulation; the yellow puncta and the red puncta represent autophagosomes and autolysosomes, respectively. The nuclei were stained by DAPI (labeled in blue). Scale bar: 5 μm. (**C**) Quantification of the autophagosomes and autolysosome puncta per cell. *n* = 3, >30 cells per experiment, mean ± SD, ** *p* < 0.01. (**D**) Immunoblotting of LC3 and p62 in HLE cells treated with TGF-β2 (10 ng/mL) and bafA1 (50 nM) for 24 h. (**E**) Quantification of the LC3 and p62 protein expression levels, normalized to the GAPDH (*n* = 3, mean ± SD * *p* < 0.05).

Previous studies and RNA sequencing have indicated that TGF-β2 has the ability to regulate autophagy. To determine the influence of TGF-β2 on autophagy flux in HLE cells, we observed the autophagic vacuoles (AVs) in TGF-β2 treated or untreated cells by transmission electron microscopy (TEM), which is an intuitively morphological examination. Under the stimulation of TGF-β2, the number of AVs markedly increased (Figure 2A), which might be caused by the activation of autophagy but also could be due to the block of autophagosome degradation. This result laid the foundation for our further evaluation of autophagic flux. Therefore, we confirmed the autophagy flux by GFP-mCherry-LC3 puncta assay and found that the number of autophagosomes (yellow) and autolysosomes (red) were both increased in TGF-β2-exposed HLE cells. In autolysosome formation, GFP signal disappearance indicated the formation of autolysosomes and only red spots were visible (Figure 2C,D), suggesting an enhanced role of TGF-β2 in autophagy flux. These findings were further verified by the protein pattern of LC3II and p62 using Bafilomycin A1, a specific V-ATPase inhibitor, which blocked the fusion of lysosomes and autophagosomes. The immunoblotting results revealed that LC3 and p62, the recognized autophagosome markers, accumulated under the BafA1 stimulation. However, in the TGF-β2 + BafA1 co-treated group, the LC3-II level was higher than in the BafA1 treated group due to the constant generation. Taken together, these results suggest that TGF-β2 may promote autophagy flux in HLE cells.

### 3.3. TGF-β2 Simultaneously Induced Autophagy and Directly Regulated TP53INP2 Expression and Subcellular Localization in HLE Cells

**Figure 3:**
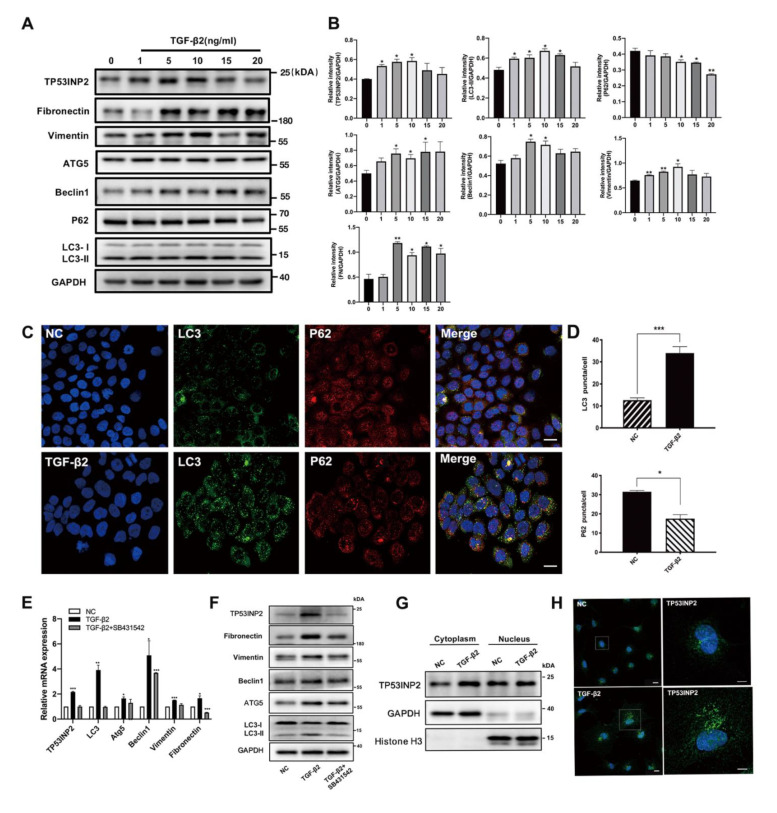
**TGF-β2 regulated TP53INP2 expression and localization during autophagy activation in HLE cells.** (**A**) Immunoblotting of TP53INP2, autophagy hallmark proteins (LC3, P62, ATG5, and Beclin1), and EMT-related proteins (fibronectin, vimentin) in HLE cells treated with different doses of TGF-β2 (0, 1, 5, 10, 15, 20 ng/mL) for 24 h. Scale bar = 10 μm. (**B**) Quantitative analysis of immunoblotting (*n* = 3, mean ± SD, * *p* < 0.05 and ** *p* < 0.01). (**C**) The localization of LC3 (green) and P62 (red) in NC and TGF-β2 (10 ng/mL)-treated HLE cells. (**D**) The number of LC3 and P62 puncta per cell in immunofluorescence experiments (*n* = 3, >30 cells per experiment, * *p* < 0.05, *** *p* < 0.001). (**E**,**F**) The mRNA and protein levels of autophagy-related proteins (LC3, ATG5, and Beclin1), EMT-related proteins (fibronectin, vimentin), and TP53INP2 are decreased in SB431542 (10 µm) and TGF-β2 co-treated cells. (**G**) Immunoblotting analysis of cytoplasmic and nuclear TP53INP2 expression level in HLE cells with or without TGF-β2 (10 ng/mL). (**H**) The localization of TP53INP2 (green) puncta in NC and TGF-β2 (10 ng/mL)-treated HLE cells. Scale bar: 10 μm.

According to the RNA sequencing, the high expression of TP53INP2 was first found in TGF-β2-treated HLE cells. To validate our hypothesis, we treated the cells with different doses of TGF-β2, ranging from 1 ng/mL to 20 ng/mL for 24 h. Western blot testing revealed that the autophagy-related protein levels of LC3-II, Atg5, and Beclin1 were increased. P62(SQSTM1), meanwhile, gradually degraded, which suggests enhanced autophagy. EMT markers, such as fibronectin (FN) and vimentin expression levels were also increased, indicating the formation of the EMT process. It is worth noting that the TP53INP2 expression level was also obviously elevated (Figure 3A,B).

We consistently observed an increased number of LC3 puncta and a reduced number of P62 puncta in TGF-β2-treated HLE cells via immunofluorescence, which verified the active autophagic response (Figure 3C,D). However, we found that the mRNA and protein levels of autophagy (LC3, ATG5, and Beclin1), EMT-related molecules (vimentin and fibronectin), and TP53INP2 were significantly increased under the stimulation of TGF-β2. While co-treated with SB431532, a specific inhibitor of TGF-β type I receptors, the activated-autophagy effect, active EMT process, and raised TP53INP2 expression level significantly declined. This suggests that TGF-β2 specifically induced autophagy and directly moderated TP53INP2 expression (Figure 3E,F). Furthermore, we explored the molecular mechanism and found that TGF-β2 promoted TP53INP2 expression through a significant increase in cytoplasmic TP53INP2, yet the levels of nuclear TP53INP2 were either unaltered or even decreased. This result was subsequently confirmed by immunofluorescence assay and it was observed that TGF-β2 could facilitate TP53INP2 movement from the nucleus to the cytoplasm to participate in autophagy-related processes (Figure 3G,H). Taken together, the above data confirm that autophagy was activated in TGF-β2-exposed HLE cells during the EMT process and that the TGF-β2 had a direct regulatory effect on TP53INP2’s expression and subcellular location.

### 3.4. Autophagy Had a Positive Regulatory Effect on the EMT Process in HLE Cells

**Figure 4:**
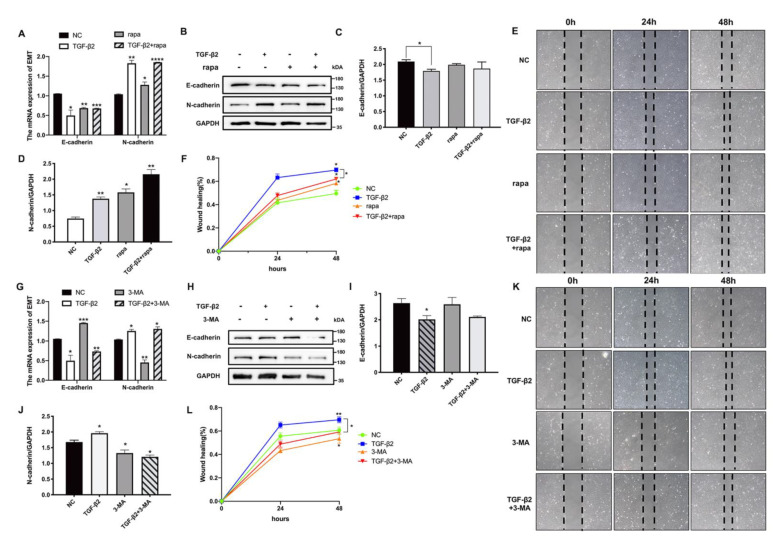
**Autophagy regulated the EMT process in HLE cells.** (**A**) The mRNA levels of the EMT markers E-cadherin and N-cadherin in TGF-β2 and rapa (200 nM) co-treated cells. Mean ± SD, *n* = 3, * *p* < 0.05, ** *p* < 0.01, *** *p* < 0.001, **** *p* < 0.0001. (**B**) The protein levels of E-cadherin and N-cadherin in TGF-β2 (10 ng/mL) and rapa (200 nM) co-treated HLE cells. (**C**,**D**) Quantitative Western blot analysis (normalized to GAPDH). Mean ± SD, *n* = 3, * *p* < 0.05, ** *p* < 0.01. (**E**) Wound healing assays were conducted on HLE cells treated with TGF-β2 and with or without rapa; the images were photographed at 0, 24, and 48 h after scratching. (**F**) Quantitative wound healing area relative to 0 h. (**G**) The mRNA levels of E-cadherin and N-cadherin in TGF-β2 and 3-MA (10 mM) co-treated cells. (**H**) The protein levels of E-cadherin and N-cadherin. (**I**,**J**) Quantitative Western blot analysis (normalized to GAPDH). Mean ± SD, *n* = 3. * *p* < 0.05, ** *p* < 0.01. (**K**) Wound healing assays of TGF-β2 and 3-MA co-treated cells. (**L**) Graph shows the percentage of the wound healing area (normalized to 0 h).

Given the dual regulatory role of autophagy on EMT in other types of cells, we examined the functional connection between autophagy and the EMT process in HLE cells. Two pharmacological agents, rapamycin (rapa) and 3-methyladenine (3-MA), were used. We co-treated the HLE cells with TGF-β2 and rapa, an activator of autophagy, which primarily blocked the MTORC1 signal pathway. As shown in Figure 4A–D, rapa effectively downregulated E-cadherin (epithelial marker) mRNA and protein levels and upregulated N-cadherin (mesenchymal marker) in HLE cells. Intriguingly, TGF-β2-treated cells and TGF-β2 + rapa co-treated cells were migrated faster than the NC group in a wound healing assay (Figure 4E,F). These results suggest that increased autophagy induced the EMT process in HLE cells. Coincidently, when autophagy inhibitor 3-MA was added to HLE cells, the opposite phenomenon was observed (Figure 4G–J): the suppression of autophagy inhibits TGF-β2-induced EMT and lowers the rate of cell migration (Figure 4K,L). Thus, the above results indicate that autophagy had a regulated effect on the EMT process.

### 3.5. TP53INP2 Is Essential for TGF-β2-Mediated Autophagy and the EMT Process

**Figure 5:**
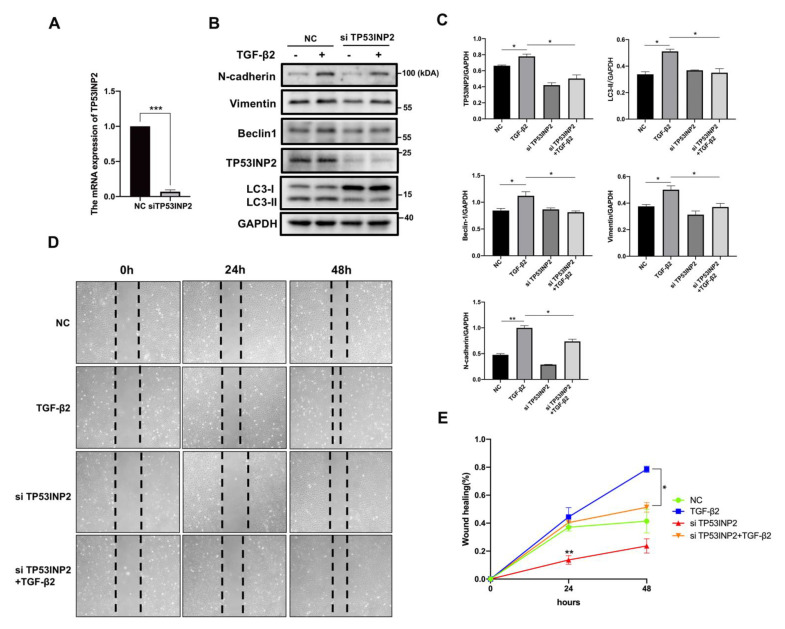
**TP53INP2 knockdown blocked the TGF-induced autophagy and the EMT in HLE cells.** (**A**) The silencing efficiency of TP53INP2 in mRNA levels. Mean ± SD, *n* = 3, *** *p* < 0.001. (**B**) Immunoblotting of N-cadherin, vimentin, Beclin1, LC3, and TP53INP2 in siTP53INP2-transfected HLE cells with or without TGF-β2 (10 ng/mL) stimulation. (**C**) Quantitative Western blot analysis (normalized to GAPDH). Mean ± SD, *n* = 3. * *p* < 0.05, ** *p* < 0.01. (**D**) Wound healing analysis. The micrographs were taken after scratching for 0, 24, and 48 h. (**E**) Quantitative wound healing area relative to 0 h. Mean ± SD, *n* = 3. * *p* < 0.05, ** *p* < 0.01.

To further investigate the functions of TP53INP2 in HLE cells, we transfected TP53INP2-specific siRNA to HLE cells and observed that the effect of RNA interference was nearly 91% (Figure 5A). The TP53INP2 knockdown decreased the autophagy-related proteins, including LC3-II conversion, as well as the Beclin1 expression. Simultaneously, N-cadherin and vimentin, two of the mesenchymal phenotype markers, were also downregulated (Figure 5B,C). This suggests that a lack of TP53INP2 abated the TGF-induced autophagic response and EMT process. The wound-healing assay also showed that the TP53INP2 siRNA group slowed down the rate of cell migration. At the same time, the co-treatment of TP53INP2 siRNA with TGF-β2 cells caused the migration rate to be slower than that of TGF-β2-treated cells (Figure 5C). Taken together, the above findings indicate that TP53INP2 played an important role in TGF-mediated autophagy and the EMT process.

## 4. Discussion

In this study, we observed the essential role of TP53INP2 in regulating TGF-β2-induced autophagy during the EMT process in HLE cells. TP53INP2, at the crossroad of autophagy and EMT in PCO pathogenesis, is likely a novel medical target for PCO therapy.

Autophagy is one of the main intracellular degradation systems for maintaining lens homeostasis [15,16]. Studies on lens-specific Atg5^−/−^ mice and Pik3c3/Vps34^−/−^ mice have discovered that impaired autophagy in lenses generates cataracts [17]. However, excessive autophagy may lead to the autophagic death of HLE cells, suggesting that the balance of autophagy is crucial for keeping the lens transparent. Given the importance of autophagy in maintaining the normal physiologic function of HLE cells, we explored the autophagic response in an abnormal state of PCO and found that TGF-β2 induced autophagy and promoted autophagy flux in HLE cells.

Further evidence has suggested that autophagy plays a fundamental role in the EMT in cancer [18]. Given the complex relationship between the two processes, we examined the influence of autophagy on EMT in HLE cells through two specific autophagy modulators, rapa and 3-MA. We observed that the activated autophagy promoted TGF-β2-mediated EMT, while blocking autophagy inhibited the EMT. 3-MA is an unspecific autophagy inhibitor and mainly blocks autophagy by inhibiting class III phosphoinositide 3-kinase to suppress autophagy induction and prevent the autophagy nucleation phase [19]. However, there are other compensatory pathways still in operation, thus the TGF-β2-induced EMT process was not completely reversed by the treatment of 3-MA. The diverse regulatory effects between autophagy and the EMT depend on different cell types and various other factors [20]. Using autophagy inhibitors as potential therapeutic targets provides a new direction for the pharmacological treatment of PCO.

TP53INP2, a nuclear protein, is highly expressed in actively metabolizing tissues and is involved in cellular processes including obesity, transcription, autophagy, and apoptosis [21,22]. Under normal conditions, TP53INP2 is predominantly localized in the nucleus and regulates transcriptional activity by binding to various nuclear hormone receptors [23]. It can also enter the nucleolus to stimulate rDNA transcription [24]. It has been reported that the translocation of LC3 from the nucleus to the cytoplasm is vital for autophagosome formation [25,26]. In response to nutrient deprivation, TP53INP2 could take LC3 to enter the cytoplasm in order to participate in phagophore development and LC3 lipidation [27,28,29]. The cytoplasmic TP53INP2 serves as a scaffold for the promotion of LC3 and ATG7 interaction and the facilitation of LC3B–PE conjugation [30].

In this study, we found that TGF-β2 could directly promote the entry of TP53INP2 into the cytoplasm and elevate TP53INP2 expression to activate autophagy and the EMT. Interestingly, the subcellular location of TP53INP2 acts as a switch to determine its biological behavior [23]. We also discovered when silencing TP53INP2 that both LC3-II conversion and the EMT process were blocked. This result was consistent with a study on bladder cancer that showed that TP53INP2 could regulate the EMT through GSK-3β signaling, supporting the idea that it has autophagy-inducing and EMT regulatory functions in HLE cells.

Given that no effective drug treatment is available, patients are still suffering from the threat of vision loss caused by PCO. Therefore, it is of great significance to find the potential drug targets. In this respect, our study provided novel insights into the interplay between autophagy and EMT induced by TGF-β2 in HLE cells and suggested that TP53INP2 could work as a molecular switch to modulate autophagy induction to fine-tune the EMT process. Of note, the role of TP53INP2 in PCO formation has not been fully clarified yet. Thus, further investigations are needed to explore the underlying mechanism of autophagy regulation by TP53INP2, and there may exist some unknown post-translational modifications and protein–protein interactions in vivo. Besides, the function of TP53INP2 may be a new target for PCO treatment.

## 5. Conclusions

In conclusion, this study focused on the role of TP53INP2 in the modulation of TGF-β2-induced autophagy and the EMT in HLE cells. During this physiological process, active autophagy is pivotal in regulating the EMT process to affect PCO development. Our findings provide new clues for understanding the pathogenesis of PCO and suggest that TP53INP2 inhibitors may be potential targets for the pharmacological treatment of PCO.

## Figures and Tables

**Table 1:** Autophagy-related genes in TGF-β2-treated HLE cells.

Gene Name	*p*-Value	Fold Change	EMT Process
RAB33A	0.02119	18.55	ND
WIPI1	0.00437	6.38	ND
MAP1LC3A	0.03464	5.24	ND
TP53INP2	0.00029	4.99	YES
TP53INP1	0.00205	4.79	YES
MTCL1	0.03377	4.65	ND
LIX1L	0.01213	4.40	ND
SRPX	0.02712	3.88	ND
SLC22A3	0.01375	3.54	YES
SMURF1	0.01375	3.08	YES
SESN2	0.01159	2.97	YES
GABARAPL1	0.01714	2.83	YES
ABL2	0.01754	2.76	YES
ULK1	0.01858	2.73	ND
TAB3	0.02837	2.71	ND
BNIP3L	0.02710	2.59	ND
QSOX1	0.03168	2.54	YES

## Data Availability

Data are available from the corresponding authors upon request.
